# Prevalence of Menthol and Menthol Capsule Cigarette Use Among 11–16 Year Olds in Wales Prior to a Ban on Characterizing Flavors in Cigarettes: Findings From the 2019 Student Health and Wellbeing Survey

**DOI:** 10.1093/ntr/ntac040

**Published:** 2022-03-11

**Authors:** Crawford Moodie, Nicholas Page, Graham Moore

**Affiliations:** Institute for Social Marketing and Health, Faculty of Health Sciences and Sport, University of Stirling, Stirling, Scotland; Centre for Development, Evaluation, Complexity and Implementation in Public Health Improvement (DECIPHer), Cardiff University, Cardiff, Wales; Centre for Development, Evaluation, Complexity and Implementation in Public Health Improvement (DECIPHer), Cardiff University, Cardiff, Wales

## Abstract

**Introduction:**

The use of flavored cigarettes has increased in many countries because of the inclusion of one or more frangible flavor-changing capsules in the filter. Research suggests that these “capsule” cigarettes appeal most to youth, but little is known about how prevalent their use is among children.

**Methods:**

A cross-sectional school survey was conducted between September and December 2019 with 11–16 year-olds (*N* = 119 388) from 198 secondary schools across Wales; the sample represented approximately two-thirds of all 11–16 year-olds in the country. The sample was asked about smoking behavior, with a quarter (*N* = 26 950) also asked about awareness of menthol cigarettes, and use of menthol and menthol capsule cigarettes if a current smoker (*N* = 1447).

**Results:**

Current smoking prevalence was 5.7% among the entire sample and 5.9% among the analytical sample (those also asked about menthol and menthol capsule cigarettes). For the analytical sample, almost all current smokers (93.2%) were aware of menthol cigarettes, with three-fifths (60.5%) reporting having used menthol cigarettes in the past 30 days (42.3% capsule cigarettes, 18.2% noncapsule cigarettes). In comparison to nonmenthol smokers, those using menthol cigarettes (capsule and noncapsule) were more likely to be frequent smokers, with those using menthol capsule cigarettes more likely to have smoked for longer.

**Conclusions:**

While past research suggests that flavored cigarettes appeal to youth, this study shows just how popular these products, and capsule cigarettes, in particular, were among young smokers in Wales. It also raises questions about why capsule cigarettes have received such limited public health attention.

**Implications:**

That three in five 11–16 year-old smokers reported using menthol cigarettes in the past 30 days highlights how appealing these products are to young people, particularly capsule cigarettes, used by seventy percent of menthol smokers. Capsule cigarettes are one of the most successful tobacco product innovations in decades, even in countries with comprehensive bans on tobacco marketing and standardized packaging. The dearth of research on capsule cigarettes is a failure of global public health. Evaluation of the ban on characterizing flavors in the United Kingdom and across the European Union is critical.

## Introduction

Reviews of the literature on menthol cigarettes show that they increase the likelihood of experimentation in comparison to nonmenthol cigarettes, with initiation with menthol cigarettes facilitating progression to established use among young smokers, and menthol cigarette smokers less successful in quitting than nonmenthol smokers.^1–3^ Subsequent research supports these findings.^4–6^ A systematic review similarly found that nonmenthol flavors help to promote and sustain tobacco use.^7^

Given the role of menthol and other flavors in encouraging smoking uptake and continued use these are now banned in cigarettes in at least 35 countries.^8^ The sharp increase in the number of countries with a flavor ban is a consequence of the “Tobacco Products Directive”,^9^ which has, since May 2020, prohibited characterizing flavors in cigarettes (factory-made or hand-rolled) across all 27 European Union (EU) countries. The ban also came into force at the same time in the United Kingdom (UK), even though it left the EU in January 2020, as the TPD had been transposed into UK law, specifically the “Tobacco and Related Products Regulations”.^10^ Prior to the ban, menthol was the only flavor permitted in cigarettes in the UK. There were at least 45 menthol cigarette brand variants on sale, which according to Japan Tobacco International had 26% of the total tobacco market.^11^ The popularity of menthol cigarettes was primarily due to the introduction, in 2011, of cigarettes with one or more rupturable capsules in the filter which could be burst, by pressing down on the filter, to change the flavor.^12^ Prior to the ban, market share of capsule cigarettes was higher in the UK than any other country in Europe and indeed most of the world.^13,14^

While capsule and traditional menthol cigarettes are now banned in the UK, it is important to explore how popular these products were among young people to provide some insight into the potential impact of the ban, and to help regulators elsewhere understand the appeal of these products to this population. While capsule cigarettes have been described by British American Tobacco as one of the most important innovations in tobacco since the filter,^15^ there remains a dearth of research on these products.^13,16^ Nevertheless, most studies suggest that capsule cigarettes are particularly appealing to youth and young adults,^17–20^ being seen as fun, cool, attractive, interactive, and allowing for customization.^21–25^ These findings offer valuable insight into why capsule cigarettes appeal to youth, but less is known about what proportion of adolescents use these products. A large school survey with 12–17 year-olds in Australia (*N* = 23 007) in 2014 found that more than half of past-month smokers reported having ever tried a capsule cigarette (51.7%), with use increasing with age and being more common among females.^26^

In this study, we explored awareness of menthol cigarettes, and recent (past 30 day) use of menthol cigarettes (capsule and noncapsule), among school children in Wales in 2019. Tobacco advertising, promotion, and sponsorship has been banned in Wales (and the rest of the UK) since July 2006, with a ban on the open display of tobacco products in retailers since April 2015. Standardized packaging for cigarettes, which includes a ban on any reference to flavor on the pack and the display of words or symbols on cigarette sticks to identify where in the filter the capsule is located, had been mandatory in Wales and across the UK since May 2017.

## Methods

### Design and Sample

The School Health Research Network (SHRN) Student Health and Wellbeing (SHW) survey, a cross-sectional online survey exploring the health and well-being of pupils in secondary schools in Wales, has been conducted biennially since 2013. The last wave, in September to December 2019, was completed by 119 388 young people aged 11–16 years, approximately two-thirds of all secondary school-aged children in Wales.

### Procedure

Schools are asked to survey all pupils or, where this is not possible, prioritize mixed-ability classes. The SHW survey is completed during school hours in a classroom setting and is designed to be administered within a single lesson. Parents are notified about the study and can withdraw their child for any reason. Participants can also opt out, and at the start of the survey are given information about the study and required to provide consent before they can participate. To maximize the number of topics which can be captured there are four versions of the survey, with a set of core common items asked of all pupils and some items asked of subsamples of pupils. All pupils are asked about tobacco use with approximately one in four asked about use of flavored tobacco.

### Measures

#### Awareness and Use of Menthol Cigarettes

To assess awareness of menthol cigarettes participants were asked: “Have you heard of cigarettes that are flavored, e.g. to taste like menthol or mint or that have a filter which can be squeezed or crushed to change the flavor?” (response options: “Yes”, “No”, “Don’t know”, “I do not want to answer”). Prevalence of menthol cigarette use (capsule and noncapsule) was assessed by asking self-reported smokers two questions taken from the ITC Youth Survey:^27^ 1) “In the past 30 days, did any of the cigarettes you smoked have a filter that you squeeze or crush for flavor?”; and 2) “In the past 30 days, were any of the cigarettes you smoked flavored to taste like menthol or mint?” (response options: “Yes”, “No”, “Don’t know”, “I do not want to answer”). A three-group measure was derived based on the response to both questions, with cigarette use classified as nonmenthol, menthol with capsule, or menthol without capsule.

#### Smoking Frequency

Smoking frequency was assessed by asking: “How often do you smoke tobacco at present?” (response options: “Every day”, “At least once a week, but not every day”, “Less than once a week”, “I do not smoke”, “I do not want to answer”). Frequency was assessed among smokers who answered both questions about use of menthol cigarettes, with use classified as daily, weekly (but not daily), or occasional (less than weekly).

#### Recency of First Cigarette

Recency of first cigarette was defined as the number of years prior to the survey since first initiating smoking, derived by subtracting age of first cigarette use from age at survey completion. Suspect cases wherein age at first cigarette use was not less than or equal to age at survey completion were omitted.

#### Socio-Demographics

Gender (“Boy”, “Girl”, “Neither word describes me”) and ethnicity (“White”, “Black, Asian and minority ethnic”) were obtained, while age was approximated using month and year of birth in combination with survey completion date. Ages falling outside the 11–16 range were omitted. The Family Affluence Scale (FAS) is a composite measure of material affluence which includes bedroom occupancy; number of household bathrooms; car, computer, and dishwasher ownership; and family holidays. It is a continuous measure with scores ranging from zero to 13, where higher scores reflect greater affluence.^28,29^

### Analysis

An analytical sample, extracted from the total sample (*N* = 119 388), consisted of all pupils asked about awareness and use of menthol cigarettes. While awareness was explored descriptively among the analytical sample, only those who reported current smoking were asked about menthol cigarette use. Single and multivariable associations between cigarette use (nonmenthol, menthol with capsule, or menthol without capsule), smoking behaviors (frequency and recency of initiation), and socio-demographic characteristics (gender, age, and ethnicity) were modeled using multinomial logistic regression, with cigarette use as the dependent variable. In all models, those reporting use of menthol cigarettes (with and without a capsule) were compared with those reporting use of nonmenthol cigarettes (the base outcome). All models were adjusted for school-level clustering, with coefficients reported as relative risk (RR) and adjusted relative risk (ARR) ratios. Missing data and “I do not want to answer” responses were omitted from the substantive analysis. All statistical analyses were undertaken using Stata v.15.1.

## Results

The analytical sample consisted of 26 950 11–16 year-olds, representing 22.6% of the total sample for the equivalent age range. Sample demographics for 11–16 year-olds are presented in Table 1. The sample was predominantly White (90.8%) with a mean age of 13.2 years. The analytical sample closely matched the total sample on several key demographics (see Table 1), including current smoking prevalence (here defined as daily, weekly, or occasional smoking), which was 5.7% (95% confidence interval [CI]: 5.6–5.8) in the total sample and 5.9% (95% CI: 5.7–6.2) in the analytical sample.

**Table 1.** Comparison of Analytical and Total Sample (Percentages and Frequencies) by Socio-Demographic Characteristics

	Analytical sample	Total sample	Regression-based *p*-value*
**Gender**% (*n*)			
Boys	48.0 (12 937)	48.7 (58 115)	.380
Girls	49.9 (13 434)	49.1 (58 610)	.319
Neither word describes me	1.1 (306)	1.2 (1472)	.215
I do not want to answer	1.0 (273)	1.0 (1,191)	.789
*Total (n)*	*26 950*	*119 388*	
**Age**mean (SD)	13.2 (1.5)	13.2 (1.5)	.895
*Total (n)*	*26 285*	*116 538*	
**White**% (*n*)	90.8 (23 632)	89.2 (103 083)	.224
*Total (n)*	*26 029*	*115 629*	
**FAS**mean (SD)	9.3 (2.4)	9.3 (2.4)	.543
*Total (n)*	*25 288*	*111 945*	
**Smoking frequency**% (*n*)			
Daily	2.6 (654)	2.8 (3127)	.243
Weekly (but not daily)	1.2 (315)	1.1 (1274)	.193
Occasional	2.2 (553)	1.8 (1995)	.002
*Total (n)*	*25 594*	*112 217*	

*Regression-based *p*-values adjusted for school clustering were estimated to test for variation in student demographics and smoking behaviors between samples. As the analytical sample is nested within the full SHW survey sample, reported *p*-values are drawn from models comparing students in the analytical sample (*n* = 26 950; 22.6%) to those in the nonanalytical (*n* = 92 438; 77.4%).

Around three-fifths (61.8%, 95% CI: 61.2–62.4) reported having heard of menthol cigarettes, while 26.0% (95% CI: 25.5–26.6) had not heard of them and 12.1% (95% CI: 11.7–12.5) were unsure. Almost all current smokers (93.2%, 95% CI: 91.8–94.4) were aware of menthol cigarettes, with three-fifths (60.5%, 95% CI: 57.9–63.0) reporting using cigarettes flavored to taste like menthol or mint in the past 30 days; 42.3% (95% CI: 39.8–44.9) capsule cigarettes and 18.2% (95% CI: 16.3–20.2) noncapsule cigarettes.

Cross-comparisons of menthol cigarette use (frequencies and percentages) with socio-demographics and smoking behaviors are presented in Table 2. These data suggest some minor variation in use of menthol cigarettes by gender, with a higher proportion of those self-identifying as neither a boy nor a girl reporting use of menthols (71.0%) compared to boys (61.5%) or girls (58.1%). Mean age by use of menthol cigarettes was similar, while a higher proportion of Black, Asian, and minority ethnic pupils reported using menthols (67.0%) relative to White pupils (59.0%). Variations according to smoking behaviors were evident, with a higher proportion of daily smokers (73.4%) using menthol cigarettes compared to weekly (64.8%) or occasional (42.0%) smokers. On average, capsule cigarette smokers reported more years smoking than noncapsule or nonmenthol cigarette smokers.

**Table 2.** Socio-Demographics and Smoking Behaviors of 11–16 Year Old Current Smokers

	Nonmenthol % (*n*)	Menthol with capsule % (*n*)	Menthol without capsule % (*n*)
**Gender** % (*n*)			
Boys	38.6 (229)	43.3 (257)	18.2 (108)
Girls	41.9 (319)	39.6 (301)	18.5 (141)
Neither word describes me (*n* = 1424)	29.0 (20)	55.1 (38)	15.9 (11)
**Age** mean (SD) (*n* = 1355)	14.3 (1.2)	14.4 (1.2)	14.2 (1.1)
**Ethnicity** % (*n*)			
White	41.0 (496)	40.1 (486)	18.9 (229)
Black, Asian & minority ethnic (*n* = 1417)	33.0 (68)	52.4 (108)	14.6 (30)
**Smoking frequency** % (*n*)			
Daily	26.6 (168)	58.2 (367)	15.2 (96)
Weekly (but not daily)	35.2 (107)	42.8 (130)	22.0 (67)
Occasional (*n* = 1447)	58.0 (297)	22.5 (115)	19.5 (100)
**Recency of first cigarette** mean (SD) (*n* = 1163)	1.3 (1.2)	2.0 (1.4)	1.4 (1.1)

Results of the multinomial regression models are presented in Table 3. In both single and multivariable models, compared to nonmenthol smokers, the relative risk for smoking menthol cigarettes with or without a capsule was greater among more frequent smokers, with the risk for smoking menthol capsule cigarettes also greater among more experienced smokers. In single-variable models, those using menthol capsule cigarettes were more likely to be older and Black, Asian, and minority ethnic in comparison to nonmenthol users. However, these associations failed to retain significance in the fully adjusted model.

**Table 3.** Single and Multivariable Multinomial Models of Menthol Cigarette Use Among 11–16 Year Old Current Smokers in Wales

	RR	ARR*
**Nonmenthol** *(base outcome)*		
**Menthol (with capsule)**		
Gender		
Boy (ref.)	**-**	-
Girl	0.84 [0.66, 1.08]	1.00 [0.76, 1.31]
Neither word describes me	1.69 [0.98, 2.92]	0.65 [0.33, 1.25]
Age	**1.12 [1.01, 1.25]**	1.02 [0.88, 1.19]
Ethnicity		
White (ref.)		
Black, Asian, & minority ethnic	**1.62 [1.21, 2.18]**	1.40 [0.86, 2.28]
Smoking frequency		
Daily	**5.64 [4.31, 7.39]**	**4.38 [3.13, 6.12]**
Weekly (but not daily)	**3.14 [2.25, 4.37]**	**3.22 [2.24, 4.62]**
Occasional (ref.)	-	-
Recency of first cigarette**	**1.46 [1.31, 1.63]**	**1.24 [1.09, 1.41]**
**Menthol (without capsule)**		
Gender		
Boy (ref.)	-	-
Girl	0.94 [0.69, 1.28]	1.01 [0.68, 1.49]
Neither word describes me	1.17 [0.54, 2.51]	1.37 [0.47, 3.93]
Age	0.95 [0.85, 1.06]	0.93 [0.79, 1.09]
Ethnicity		
White (ref.)	-	-
Black, Asian, & minority ethnic	0.96 [0.63, 1.46]	0.84 [0.49, 1.43]
Smoking frequency		
Daily	**1.70 [1.19, 2.41]**	**1.75 [1.14, 2.69]**
Weekly (but not daily)	**1.86 [1.23, 2.82]**	**1.93 [1.24, 2.99]**
Occasional (ref.)	-	-
Recency of first cigarette	1.07 [0.96, 1.20]	1.03 [0.89, 1.20]

*fully adjusted model, *n* = 1143;

^**^ number of years prior to survey (zero to 5+ years); ***p* < .05**

## Discussion

In this nationally representative school sample in Wales, among smokers asked about menthol cigarette use (approximately a quarter of the total sample) three-fifths reported having used these products in the past 30 days, with most (70%) using menthol capsule cigarettes. Those using menthol cigarettes were more likely to be frequent smokers than those using nonmenthol cigarettes, with almost three-quarters of daily smokers having smoked menthol cigarettes in the past 30 days. Menthol capsule smokers were also more likely to have smoked for a longer period of time. These findings are consistent with reviews that suggest that menthol cigarettes facilitate progression to established use among young smokers.^1–3^

While only a quarter of the (albeit very large) sample was asked about menthol use, there is no obvious reason to suspect that the findings for the total sample would have been notably different, given that smoking prevalence and the socio-demographic profiles of the total and analytical samples were comparable and the literature suggests menthol and capsule cigarettes are viewed positively by youth.^13,16^ We are unable to provide any insight into the type of cigarette first used, however, and therefore whether menthol or menthol capsule cigarettes appear to be starter products.^1–3^ Nor can we offer any insight into why young smokers were more likely to use capsule cigarettes than nonmenthol or noncapsule menthol cigarettes, although previous research suggests it is because they are viewed as fun, cool, attractive, interactive, and allow for customization.^21–25^ Nevertheless, the findings suggest that menthol and particularly menthol capsule cigarettes may help to sustain smoking.

**Figure 1.**
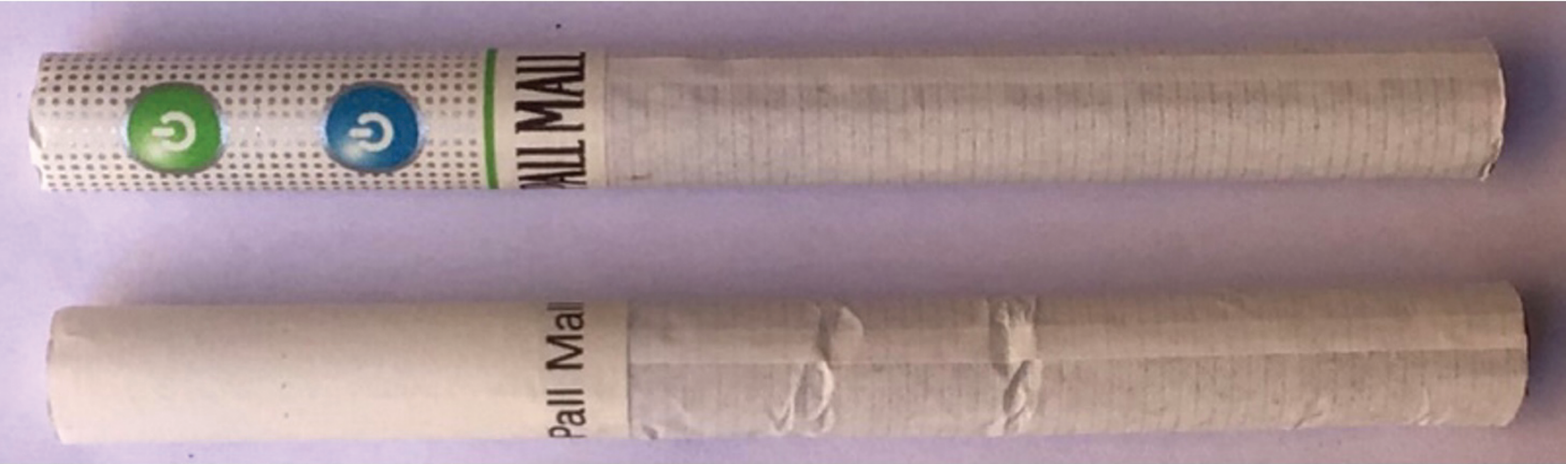
Capsule cigarettes before (top) and after standardized packaging.

In Wales, and the rest of the UK, standardized packaging has been mandatory for cigarettes (factory-made or hand-rolled) since May 2017. Evaluative research suggests that it has reduced pack appeal and increased the salience of the on-pack warnings, and appears to be discouraging youth from smoking.^30^ Standardized packaging also makes it more challenging for tobacco companies to promote their products. For menthol cigarettes in the UK (and countries in the EU with standardized packaging) this challenge is even more pronounced as any reference to flavor on packs, and any words or symbols on the filter to identify where a capsule is located (see Figure 1), is not permitted. This has not discouraged tobacco companies from increasingly focusing on filter innovation in the UK, as they have in other markets with standardized packaging,^31–34^ with several new capsule cigarette variants introduced poststandardized packaging,^35^ including at least five in 2018.^13^ Our findings suggest that even in countries where all tobacco marketing is banned (including the open display of tobacco products in tobacco-selling retailers) and standardized packaging is required, policies intended primarily to safeguard young people from tobacco companies’ promotional practices, this is insufficient to protect children from tobacco product innovation. In such markets, future research exploring young peoples’ awareness of tobacco product innovations, and how they become aware of these, would be of value.

The popularity of menthol and capsule cigarettes among youth helps to justify the flavor ban in the UK and across the EU. It raises a question as to why capsule cigarettes were almost completely overlooked by public health. It was estimated, in 2013, that over 207 000 children aged 11–15 years old start smoking each year in the UK,^36^ and while these figures will have declined in parallel with a reduction in youth smoking prevalence, it is likely that over one million young people in the UK started smoking since these products entered the market in 2011. Even a very conservative estimate would suggest that tens of thousands started or continued smoking with capsule cigarettes. The focus on e-cigarettes may explain why capsule cigarettes has failed to capture the attention of those working in public health in the UK,^22^ but to neglect a product that makes cigarettes cool and fun to children^13^ and that is clearly their product of choice, as demonstrated in this study, is a significant failing.

Evaluation of the flavor ban in the UK, and across the EU, is imperative. The only country wide flavor ban to have been evaluated is in Canada.^37–46^ This evaluation provides valuable insight into the impact of the ban, but in Canada menthol cigarettes had low market share (<5%), capsule cigarettes were introduced after the legislation was announced, and no study explored the response of young people. In addition, consumers and the tobacco industry may respond differently in the UK for several reasons: 1) The ban in Canada covers all tobacco products (except cigars of a certain size) whereas in the UK it only covers cigarettes and rolling tobacco, and excludes tobacco accessories, which offers consumers choice and the tobacco industry opportunities, 2) E-cigarettes are promoted for harm reduction in the UK, whereas they were not in Canada, thus flavored e-cigarettes (as well as flavored heated tobacco products) may be a more popular substitute for menthol smokers, 3) Tobacco companies in Canada informed consumers about the menthol ban and promoted replacement nonmenthol variants via the packaging, which was not possible in the UK because of standardized packaging, 4) Menthol cigarettes are legally available in First Nations Reserves in Canada (exempt from the legislation), and while sale is restricted to First Nations people this contrasts with the UK (and EU) where there is no legal source, and 5) The UK menthol ban took effect in the midst of a pandemic, which may have had a range of impacts, such as influencing retailer compliance.

In terms of limitations, while assurances were given to school pupils that the survey was anonymous and their responses would not be shared without their permission, as is common with self-report data, we cannot reject the possibility of social desirability bias. As the survey was completed during school class time, it is possible that this may have affected pupil responses with respect to smoking. Nevertheless, menthol cigarettes were clearly popular among current smokers in this sample. Although prevalence of use of menthols was measured among a large subsample of survey respondents, we did not measure frequency of use. Nor were we able to provide insight into “dual users”, i.e. those using both menthol and capsule cigarettes, because those responding “Yes” to the two questions asking whether their cigarette has a capsule, and whether it tastes like menthol or mint, may be dual users or exclusive capsule smokers. This may be an area for future research.

This study shows how popular menthol cigarettes, and particularly menthol capsule cigarettes, are among youth smokers in Wales. Considerably more research on, and attention to, capsule cigarettes is clearly warranted. Comprehensive evaluation of the flavor ban in place in the UK and in EU countries is essential to understand whether the policy has met its objectives (e.g. increased cessation among menthol smokers, reduced uptake) and any unintended consequences (e.g. the growth in sales of illicit menthol products, use of aftermarket products to flavor traditional cigarettes). It is also important for policy adoption in other markets.

## Supplementary Material

A Contributorship Form detailing each author’s specific involvement with this content, as well as any supplementary data, are available online at https://academic.oup.com/ntr.

ntac040_suppl_Supplementary_Taxonomy_Form

## Data Availability

Data from the Student Health and Wellbeing Survey are available upon reasonable request and abidance with the School Health Research Network’s Data Use Protocol. Further information is available from shrn@cardiff.ac.uk.
